# Comparing Backward Walking Performance in Parkinson’s Disease with and without Freezing of Gait—A Systematic Review

**DOI:** 10.3390/ijerph20020953

**Published:** 2023-01-04

**Authors:** Tracy Milane, Clint Hansen, Matthias Chardon, Edoardo Bianchini, Nicolas Vuillerme

**Affiliations:** 1 AGEIS, Université Grenoble Alpes, 38000 Grenoble, France; 2 Department of Neurology, UKSH Campus Kiel, Kiel University, Arnold-Heller-Str. 3, Haus D, 24105 Kiel, Germany; 3 Department of Neuroscience, Mental Health and Sensory Organs (NESMOS), Sapienza University of Rome, 00189 Rome, Italy; 4 LabCom Telecom4Health, Orange Labs & Université Grenoble Alpes, CNRS, Inria, Grenoble INP-UGA, 38000 Grenoble, France; 5 Institut Universitaire de France, 75005 Paris, France

**Keywords:** Parkinson’s disease, freezing of gait, backward walking, gait

## Abstract

Introduction: Parkinson’s disease (PD) is a neurodegenerative disease characterized by motor symptoms and gait impairments. Among them, freezing of gait (FOG) is one of the most disabling manifestations. Backward walking (BW) is an activity of daily life that individuals with PD might find difficult and could cause falls. Recent studies have reported that gait impairments in PD were more pronounced in BW, particularly in people presenting FOG. However, to the best of our knowledge, no systematic review has synthetized the literature which compared BW performance in PD patients with and without FOG. Objective: The aim of this study was to evaluate the differences in BW performance between PD patients with FOG and PD patients without FOG. Methods: Two databases, PubMed and Web of Science, were systematically searched to identify studies comparing BW performance in PD patients with and without FOG. The National Institutes of Health (NIH) tool was used to assess the quality of the studies included. Results: Seven studies with 431 PD patients (179 PD with FOG and 252 PD without FOG) met the inclusion criteria and were included in this review. Among them, 5 studies reported walking speed, 3 studies step length, stride length and lower limb range of motion, 2 studies functional ambulation profile, toe clearance height, swing, and stance percent and 1 study reported the decomposition index and stepping coordination. Compared to PD patients without FOG, PD patients with FOG showed slower walking speed and reduced step length in 3 studies, shorter stride length, lower functional ambulation profile and decreased ankle range of motion in 2 studies, and smaller swing percent, higher stance percent, worse stepping coordination, greater decomposition between movements, and lower toe clearance height in one study. Conclusion: Despite the small number of included studies, the findings of this review suggested that PD patients with FOG have worse gait performance during the BW task than PD without FOG.

## 1. Introduction

Parkinson’s disease (PD) is a progressive neurodegenerative disorder [1] characterized by motor symptoms such as slow movement, muscle stiffness, and tremor affecting the performance of motor tasks, including walking [2]. Gait impairments are rather common in PD, particularly with disease progression [3] and are associated with an altered quality of life and an increased risk of falls [4]. These include continuous alterations such as reduced step length, low gait speed, increased cadence and variability, and episodic disturbances such as freezing of gait (FOG) [3]. FOG is a debilitating symptom of PD [5], defined as a “brief, episodic absence or marked reduction of forward progression of the feet despite the intention to walk” [6]. It impairs mobility, alters quality of life, leads to falls and is most likely to occur when initiating gait, turning, walking through narrow spaces, or approaching a destination [6].

Mobility in PD could be assessed using different tasks such as forward walking, backward walking (BW), sit-to-walk, and turning [7]. Forward walking is a basic component in human locomotion and BW is a variation of it [8]. BW is also a common activity of daily living, performed, for instance, when stepping back from a sink or when a fast-moving bus passes [9]. However, BW is more difficult and demanding because of increased postural instability and absence of visual cues [10] and elderly people demonstrated reduced cadence, shorter stride length and swing phase, and increased double support time during BW [11]. Since BW is an essential element of mobility, deficits might lead to higher risk of falls. To this end, the evaluation of BW performance could be clinically useful [12] and BW has been reported to be more accurate than forward walking in identifying the risk of falls in elderly people [13].

Concerning individuals with PD, these patients demonstrated more pronounced gait impairments in BW than in forward walking such as reductions in stride length and velocity [14,15]. In addition, in PD, BW impairment, differently from forward walking, has been reported to be non-responsive to levodopa, suggesting that this paradigm may provide further information about mobility [13]. Finally, BW alterations in PD have been reported to be more closely related to motor symptoms and fear of falling than forward walking, thus suggesting that BW analysis could be a potential progression biomarker [16]. Recent studies have also reported that patients with PD, particularly those with FOG, had greater impairments in BW than in forward walking [7,9,17,18,19,20,21]. This subgroup of patients demonstrated to be more prone to multiple falls and injuries [22] and backward walking has been linked to FOG occurrence [18]. Therefore, the implementation of BW evaluation in PD patients reporting FOG could be particularly relevant and could add valuable information to clinical assessment. However, to the best of our knowledge, no systematic review to date has compared BW performance reported in PD patients with and without FOG. Thus, the aim of this review was to evaluate the differences in BW performance between PD patients with FOG and PD patients without FOG.

## 2. Methods

### 2.1. Protocol and Registration

This systematic review’s protocol has been registered with the International Prospective Register of Systematic Reviews (PROSPERO) (CRD42022354994). The review follows the recommendations of the Preferred Reporting Items for Systematic Reviews and Meta-Analyses (PRISMA) statement [23] and the Cochrane Handbook for Systematic Reviews guidelines [24]⁠.

### 2.2. Eligibility Criteria

This review included original articles published in peer-reviewed scientific journals. Only articles published in English, French, Italian or German were included. Participants had to be diagnosed with PD. Studies reporting any measure quantifying BW performance (spatiotemporal parameters, kinematic parameters, etc.) and comparing PD patients with and without FOG were included. Case reports, abstracts, editorials, letters to the editor, case studies, reviews, and meta-analyses were excluded.

### 2.3. Data Sources and Search Strategy

The following two databases, PubMed and Web of Science, were systematically searched on 4 July 2022, and the search was repeated on 6 November 2022.

Keywords related to (1) Parkinson’s disease, (2) backward walking, and (3) freezing of gait have been used. The search strategy consisted of a combination of keywords using the Boolean operator “AND” and “OR”. The first category included terms related to PD such as “idiopathic Parkinson’s Disease” OR “Lewy Body Parkinson Disease” OR “Primary Parkinsonism” OR “Idiopathic Parkinson Disease” OR “Parkinson Disease”. The second category focused on terms related to backward walking such as “backwards walking” OR “backward walking” OR “backward gait” OR “backward locomotion” OR “backwards locomotion” OR “retrowalking” OR “retro-walking”. The third category specified FOG and comprised the following terms: “freezing of gait” OR “fog” OR “frozen gait”. For the final search, these 3 keyword’s categories were combined as follows: (1) AND (2) AND (3). Search fields were restricted to the abstract, title, and keywords.

### 2.4. Study Selection

Two independent reviewers (TM and MC) screened the titles and abstracts of all studies to identify potentially relevant articles. After the removal of duplicates, selected full-text articles were then reviewed and independently screened for eligibility according to the inclusion criteria mentioned above. If disagreements occurred between the two independent reviewers, consensus will then be achieved through discussions or contact with a third reviewer (NV) to arbitrate the disagreement.

### 2.5. Data Extraction

After the end of the selection process, two independent reviewers (TM and MC) extracted data from all included studies. Information was extracted on (1) the study’s characteristics, (2) the sample characteristics, (3) the measure of FOG, (4) the measure of BW, and (5) the main results of the comparison of BW performance in PD patients with and without FOG. Reviewers were not blinded to the authors or journals when extracting data. Any discrepancies between these independent reviewers were resolved at a consensus meeting. If disagreement persisted, a third reviewer (NV) was consulted for a final decision.

### 2.6. Methodological Quality

Two independent reviewers (TM and MC) assessed the quality of the included studies. The National Institutes of Health (NIH) Quality Assessment Tool for Observational Cohort and Cross-sectional Studies (NIH, 2014) was applied to assess the methodological quality of the included studies. It consists of answering yes, no, or other to each of the 14 items. Then, the overall quality of a study was evaluated by assigning a good, fair, or poor rating to each study. Any disagreements between the two independent reviewers were resolved by discussion, with the involvement of a third reviewer (NV) when necessary, who was contacted to arbitrate the disagreement to reach the final rating.

## 3. Results

### 3.1. Study Selection

**Figure 1:**
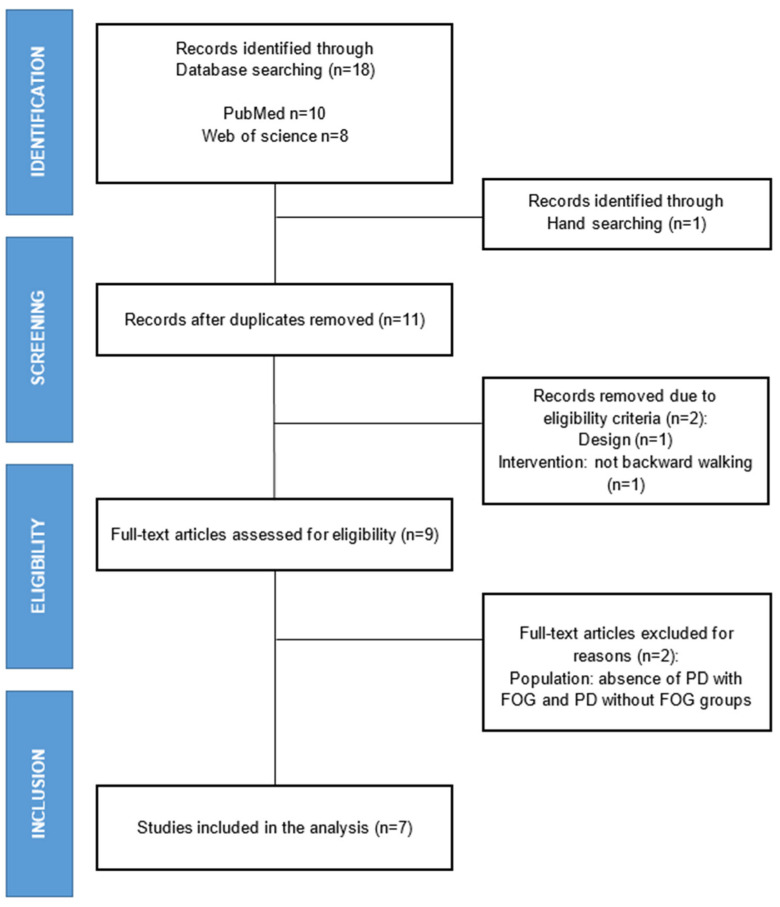
Flowchart of the study selection process.

The database search yielded 18 potentially relevant studies. One additional study was identified through hand searching. After removing duplicates (*n* = 8), 11 studies remained. After the screening of titles and abstracts, 2 studies were excluded, and 9 studies were reviewed for eligibility. After full-text screening, 2 studies were excluded. The remaining 7 studies fulfilled the eligibility criteria and were included in this systematic review [7,9,17,18,19,20,21]. The study selection process is illustrated in Figure 1.

### 3.2. Methodological Quality

The overall quality assigned to the 7 studies was “fair” according to the NIH tool (Table 1).

### 3.3. Study Characteristics

Table 2 shows general information about the studies included in this systematic review, including authors, year of publication, country, title, journal, and funding sources.

Publication year. Publication year of the 7 included studies ranged from 2009 [9] to 2022 [7]. One study (14%) was published in each of the following years: 2009 [9], 2010 [17], 2012 [18], 2017 [19], 2018 [20], 2020 [21], and 2022 [7].

Country of the first author’s affiliation. Five studies (71%) were conducted in the USA [9,17,18,19,20} and 2 (29%) in the Republic of Korea [7,20].

Journal. The 7 included studies have been published in 6 different journals: 2 (29%) in *Gait & Posture* [7,20], and 1 (14%) in each of the following journals: *Movement Disorders* [9], *Neurorehabilitation and Neural Repair* [17], *Journal of Applied Biomechanics* [21], *Parkinsonism and Related Disorders* [18] and *Journal of Rehabilitation Medicine* [19].

Funding. Funding information was reported in all studies (*n* = 7). Four studies (57%) were supported by the American Parkinson Disease Association in the USA [9,17,18,21]. Four studies (57%) received grants from the National Institutes of Health [17,18,19,21]. One study (14%) was supported by the Parkinson’s Disease Foundation in USA [18]. Two studies (29%) were supported by the Dong-A University research fund in the Republic of Korea [7,20].

Design. Six studies (86%) [9,17,18,19,20,21] were cross-sectional analyses and one (14%) was explorative [7].

### 3.4. Sample Characteristics

Table 3 shows basic demographic and anthropometric information of the participants included in each study.

The 7 studies included covered a total of 431 patients with PD, among whom 248 (57%) were male. All studies included both men and women participants.

In 2 studies (29%) [7,20], patients were diagnosed with idiopathic PD according to the United Kingdom Brain Bank criteria [25,26], while in 3 studies (43%) [9,17,19], PD was determined using criteria for clinically defined “definite PD” [27,28]. Two studies (29%) did not mention how PD was diagnosed [18,21].

The mean PD sample size was *n* = 31 ± 16, ranging from 10 [20] to 65 [19] patients with PD. The mean PD age was 68.6 ± 1.7 years, ranging from 64.2 ± 6.6 [21] to 72 ± 9 years [18]. The average total score of the Movement Disorder Society-Unified Parkinson’s Disease Rating Scale (MDS-UPDRS) was reported in 4 studies [7,9,17,20] and ranged from 26.2 ± 8.7 [17] to 51.80 ± 14.47 [20]. The average MDS-UPDRS part III score (motor examination) was reported in 5 studies [7,9,17,18,20] and ranged from 27.5 ± 9.2 [17] to 45.5 ± 15.2 [18]. The mean Hoehn and Yahr (H&Y) score was reported in 3 studies [7,18,20] and ranged from 2.23 ± 0.59 [20] to 2.63 ± 0.83 [18]. Mean disease duration was reported in 3 studies [9,17,18] ranging from 6.4 ± 3.7 [17] to 10.5 ± 5.9 years [17]. Symptom duration was reported in 2 studies [7,20] ranging from 4.5 ± 4.0 [7] to 8.1 ± 5.1 years [7].

All 7 of the included studies [7,9,17,18,19,20,21] classified the PD population into 2 categories according to the absence (PD − FOG) (*n* = 252 participants, 58%) or presence (PD + FOG) of FOG (*n* = 179 participants, 42%). In 3 studies, PD patients were considered in PD + FOG group with a score > 1 [9,17] or a score > 2 [18] on item 3 of the Freezing of Gait Questionnaire (FOGQ) (“Do you feel that your feet get glued to the floor while walking, making a turn or when trying to initiate walking (freezing)?”). In 4 studies [7,19,20,21], PD patients were considered in PD + FOG group according to their responses to the new Freezing of Gait Questionnaire (NFOG-Q). Patients watched a video representing diverse types of FOG and then were grouped in PD + FOG group if they answered positively to item 1 of the NFOG-Q (“Did you experience ‘freezing episodes’ over the past month?”).

#### Studies Population

The 7 studies included covered a total of 179 PD patients (42%) with FOG (PD + FOG) and 252 PD patients (58%) without FOG (PD − FOG). Details about included studies, sample size, demographic, and anthropometric and clinical characteristics are shown in Table 3.

### 3.5. Assessments of Freezing of Gait

Two methods were used to assess the presence and severity of FOG in patients with PD: Freezing of Gait Questionnaire (FOG-Q) [29] and the new Freezing of Gait Questionnaire (NFOG-Q) [30].

Method 1. FOG-Q [29] was used in 3 studies (43%) [9,17,18], including a total of 187 participants, namely 105 PD without FOG (56%) and 82 PD with FOG (44%). The FOG-Q is a self-administrative scale which consists of 6 items, each item scored from 0 to 4 with a maximum score of 24 points. A higher score represents more severe freezing. Four items question the duration and frequency of FOG, and two items question general gait impairment. Participants were considered in the PD + FOG group, if they had a score > 1 on item 3 (“Do you feel that your feet get glued to the floor while walking, making a turn or when trying to initiate walking (freezing)?”), suggesting a frequency of FOG at least once a week [29].

Method 2. NFOG-Q [30] was used in 4 studies (57%) [7,19,20,21], including a total of 244 participants, namely 147 PD without FOG (60%) and 97 PD with FOG (40%). The NFOG-Q was based on the FOG-Q version. An initial part was added to enable the detection of FOG, and the exclusion of subjects without symptoms, from the actual score of FOG severity and impact. A short video was also added to help in clarifying diverse types and durations of FOG. Participants were considered in the PD + FOG group if they answered “yes” on item 1 (“Did you experience freezing episodes over the past month?”) [30].

### 3.6. Backward Walking Protocol

Table 4 reports description of the BW tasks.

#### 3.6.1. Experimental Procedure

Instructions. In one study (14%) [9], participants were instructed to walk at their normal pace, 3 times in each direction: forward then backward. In one study (14%) [17], participants performed single-task conditions at their normal or comfortable pace 3 times in each direction: forward then backward. Secondly, they performed one trial of a mental arithmetic task with forward then backward. In 2 studies (29%) [18,21] participants were asked to walk backward at a comfortable pace. In 3 studies (43%) [7,19,20], the instructions for the tasks were not reported.

Walking speed. Participants’ walking speed was reported in 6 studies (86%): normal speed in 3 studies (43%) [9,17,20], comfortable speed in 2 studies (29%) [18,21] or preferred speed in one study (14%) [18].

Walking distance. Walking distance was reported in all studies. Participants walked a length of 10 m [18], 8 m [7,20], 7 m [21], 5 m [9,17] and 4.8 m [19].

Number of trials. In 5 studies (71%), participants performed 3 trials per condition [7,9,17,19,20]. In one study (14%), they performed 5 trials [21] and in one study (14%) they performed 5 to 8 trials [18]. Only Hackney and Earhart (2010) reported that no practice trials were given to participants. In 3 studies (43%), participants were given enough time to rest and were allowed to sit between trials [9,17,19]. Results from trials were averaged in 5 studies (71%) [7,9,17,19,20], and it was not mentioned whether results were averaged or not in 2 studies (29%) [18,21].

Medication state during task. BW was assessed in people with PD in OFF-medication state, with assessments being carried out after not taking antiparkinsonian medication at least 12 h before the tests in 4 studies (57%) [7,18,19,20] (*n* = 231 participants) and in the ON-medication state in 3 studies (43%) [9,17,21] (*n* = 200 participants).

There were no included studies in which participants were asked to perform BW only. In two studies (29%) [9,20] participants were asked to perform two walking tasks: backward and forward walking. In Hackney and Earhart’s (2009) study, participants walked forward then backward. The order of presentation of the conditions was not reported by Son et al. (2018).

In one study (14%) [21], participants were asked to perform three walking tasks: BW, forward walking, and forward walking with a dual task (listing as many words as they could think of that start with a certain letter). By condition, trials were grouped into blocks, five trials per block, and the blocks were randomized.

In one study (14%) [17] participants were asked to perform four walking tasks: backward and forward walking in single- and dual-task conditions (performing a mental arithmetic task aloud that consisted of counting backward from 100 by threes, from 50 by fours, and from 75 by sixes). Participants walked forward then backward.

In one study (14%) [18], participants were asked to perform 6 walking tasks: BW, forward walking, turning right and left in a small radius circle and turning right and left in a large radius circle. These 6 walking tasks were performed in random order.

In one study (14%) [7], participants were asked to perform 4 walking tasks: BW, forward walking, and 360-degree turning in both directions. The order of presentation of the conditions was not reported.

In one study (14%) [19], participants were asked to perform 4 walking tasks: BW, forward walking, MDS-UPDRS-III item 12 (for postural responses), and the Mini Balance Evaluation Systems Test items 4 and 5 (for postural responses). The order of presentation of the conditions was not reported.

#### 3.6.2. Data Acquisition System

Table 5 describes the data acquisition method and measured parameters during BW.

BW was captured using cameras in three studies (43%): in two studies (29%) (Son et al., 2018; Son et al., 2022) six infrared cameras (Vicon, MX-T10, Bilston, UK) were used; in one study (14%) (Myers et al., 2020) a Hawk Digital RealTime 8-camera system (Motion Analysis Corp, Santa Rosa, CA, USA) with a 3.048 m × 3.048 m × 3.048 m capture volume and 100-Hz capture rate were used.

In one study (14%) (Peterson et al., 2012), to track the moment of heel strike and toe off, each shoe’s sole had six round footswitches (20 mm (about 0.79 in) in diameter and 1 mm (about 0.04 in) thick; Motion Lab Systems, Baton Rouge, LA, USA) attached to it.

### 3.7. Comparison of BW Performance in PD Patients with and without FOG

Spatiotemporal parameters were measured in all studies (*n* = 7) [7,9,17,18,19,20,21]. Three studies (43%) [7,20,21] measured both spatiotemporal and kinematic parameters.

Five studies (71%) [7,9,17,19,20] reported walking speed (m/s).

Three studies (43%) [7,19,20] reported step length (m). Three studies (43%) [7,9,17] reported stride length (m). Three studies (43%) [7,9,17] reported cadence (step/min).

Two studies (29%) [7,20] reported step time (s) and asymmetry index of step time and length. Two studies (29%) [9,17] reported swing and stance percent, base of support (m), and functional ambulation profile which quantify gait variability. One study (14%) [7] reported stride time (s). One study (14%) [17] reported gait asymmetry. The other study (14%) of Hackney and Earhart reported double support percentage [9]. One study (14%) [18] reported stepping coordination measured as phase coordination index, a temporal gait variable that quantifies the accuracy and consistency of left to right stepping phases by assessing bilateral coordination of gait. It was calculated as the summation of 2 components: consistency of phase generation and temporal accuracy in producing anti-phased stepping through all steps [18]. One last study (14%) [21] reported cycle timing, % gait cycle of the hip, and knee and ankle joints measuring the timing of maximum and minimum angles.

Kinematic outcomes computed during BW in individuals with PD with and without FOG are presented in Table 6.

Kinematic parameters were measured in 3 studies (43%) [7,20,21], which reported the range of motion (ROM) of the hip, knee, and ankle joints.

Two studies (29%) [7,20] reported the maximum anti-phase (°) and toe clearance height (cm).

One study (14%) [21] measured the decomposition index which was calculated to determine percentage of the gait cycle in which a joint remained fixed while another one is moving.

#### 3.7.1. Spatiotemporal BW Parameters

The distribution of the kinematics and spatiotemporal parameters in included studies was reported in Table 6.

Spatiotemporal outcomes during BW in individuals with PD with and without FOG were summarized in Table 5.

Walking speed. In three studies (43%) [17,19,20], walking speed was significantly slower in PD + FOG compared with PD − FOG.

In one study (14%) [20], walking speed in PD + FOG was significantly slower by 23% in both the more affected side (MAS) and the less affected side (LAS) compared with PD − FOG (MAS and LAS: 0.46 ± 0.13 and 0.46 ± 0.13, respectively, in PD + FOG; 0.60 ± 0.14 and 0.60 ± 0.14, respectively, in PD − FOG; *p* < 0.05) [20]. The median walking speed was also lower by 26% in PD + FOG compared with PD − FOG (*p* < 0.05) in another study [19]. In addition, PD + FOG walked slower than PD − FOG (*p* < 0.00625) in both single- and dual-task conditions [17].

**Figure 2:**
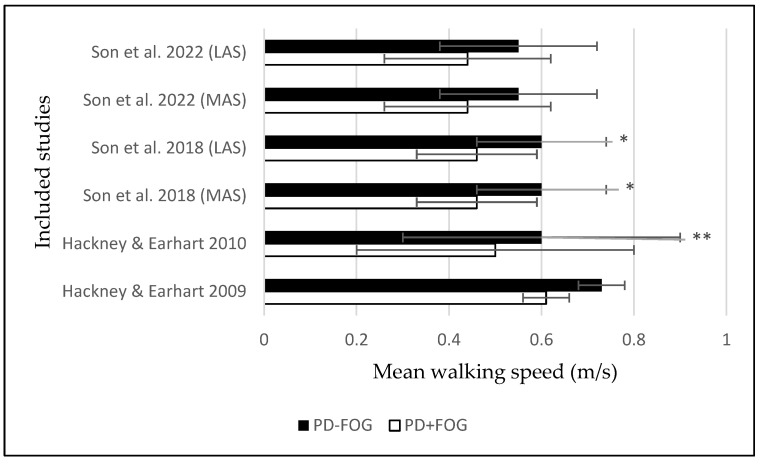
Mean and standard deviation of the mean walking speed obtained in Parkinson’s disease patients with and without freezing of gait during backward walking. The 2 groups are presented as: Parkinson’s disease patients without freezing of gait (black bars) and Parkinson’s disease patients with freezing of gait (white bars). The significant *p* values for comparison between the two groups are reported: (*: *p* < 0.05, **: *p* < 0.01) [7,9,17,20].

Figure 2 illustrates the average walking speed measured in PD patients with and without FOG.

Step length. In 3 studies (43%) [7,19,20] step length was significantly lower in PD + FOG than PD − FOG.

PD + FOG group had significantly narrower step length in the most affected side than PD − FOG group ([20]: 0.22 ± 0.08 vs. 0.32 ± 0.09, *p* = 0.014; [7]: 0.25 ± 0.11 vs. 0.30 ± 0.07, *p* < 0.05) [7,20]. Also, the median step length was smaller by 28% in PD + FOG compared with PD − FOG (*p* < 0.05) [19]. PD + FOG had an increased asymmetry of step length (24.03 ± 19.20 vs. 11.03 ± 7.62, *p* = 0.030) [20].

Stride length. In two studies (29%) [9,17], PD + FOG had shorter stride length by 12.5% than PD − FOG ([9]: 0.7 ± 0.04 vs. 0.8 ± 0.05, *p* = 0.032). In both simple- and dual-task BW, PD + FOG walked with shorter strides [17].

Cadence. In 3 articles (43%) [9,17,20], there was no significant difference in cadence between PD + FOG and PD − FOG groups.

Functional ambulation profile. PD + FOG had significantly lower functional ambulation profile by 13% than PD − FOG ([9]: 55.8 ± 2.2 vs. 64.1 ± 2.5, *p* = 0.027). In both simple- and dual-task BW, PD + FOG had lower functional ambulation profile values compared with PD − FOG [17]. In two studies (29%) [9,17], PD + FOG had a lower functional ambulation profile than PD − FOG, by 13% in Hackney and Earhart 2009: 55.8 ± 2.2 vs. 64.1 ± 2.5, *p* = 0.027).

Swing and stance percent. PD + FOG had significantly smaller swing percent by 6% and greater stance percent by 3% than PD − FOG (30.3 ± 0.8 vs. 32.4 ± 0.6, *p* = 0.040 and 70.0 ± 0.9 vs. 67.7 ± 0.6, *p* = 0.031, respectively) [9]. PD + FOG were more variable in BW swing and stance percent than PD − FOG (swing %: 8.6 ± 3.2 vs. 6.4 ± 2.8, *p* = 0.013; stance %: 10.8 ± 3.0 vs. 5.7 ± 0.5, *p* = 0.010) [9].

Phase coordination index. PD + FOG had significantly higher phase coordination index values than PD − FOG, suggesting worse stepping coordination in PD + FOG (13.9 ± 3.9 vs. 10.9 ± 3.8) [18].

Cycle timing, % gait cycle. For the PD + FOG, the minimum ankle angle happened late in the gait cycle compared with the PD − FOG (95.2 ± 5.2 vs. 88.3 ± 8.4) [21].

#### 3.7.2. Kinematic BW Parameters

Range of motion. PD + FOG group had significantly decreased ROM in the ankle joint in the most affected side [7,20] ([20]: 21.84 ± 5.60 vs. 30.43 ± 6.75; [7]: 21.70 ± 6.60 vs. 28.00 ± 7.40) and the less affected side, (21.18 ± 3.31 vs. 27.67 ± 4.84) [20] and hip joint in the less affected side (25.77 ± 7.39 vs. 33.76 ± 9.25) [20] than PD − FOG group.

Toe clearance height. PD + FOG had a lower toe clearance height in the most affected side than PD − FOG group (2.10 ± 0.65 vs. 2.96 ± 0.94) [20].

Decomposition indices. Greater decomposition indices between hip–ankle and hip–knee movements were observed in the PD + FOG group (12.9 ± 8.2 vs. 8.8 ± 4.1 and 16.0 ± 8.3 vs. 10.2 ± 6.5, respectively) [21].

## 4. Discussion

To the best of our knowledge, this is the first systematic review summarizing published studies that compared BW performance in PD patients with and without FOG. The main findings of our review suggest that PD + FOG exhibited marked differences in BW compared to PD − FOG. PD + FOG had a slower walk with shorter strides and steps and a lower functional ambulation profile compared with PD − FOG. Furthermore, PD + FOG had worse stepping coordination, reduced swing percent, and greater stance percent. They also had a decreased ROM in the ankle and hip joints, greater movement decomposition and lower toe clearance height than PD − FOG. These findings could be explained by a reduction in proprioception, an impairment in attention and cognitive functions, or an alteration in visuospatial and cerebellar network in PD + FOG patients.

BW requires greater proprioception than forward walking due to the lack of visual control. In PD patients, an alteration of proprioception has been described [31] and, consequently, individuals with PD are more dependent on visual information [32] which is more pronounced in those with FOG [33]. This could explain why PD patients have greater impairments while walking backward [17] and the marked differences between PD with and without FOG [20].

Another factor that could explain our findings is the increased cognitive demand for BW. BW is a complex task, demanding more investment in attention and cognitive resources. As reported previously, individuals with PD might experience cognitive impairment, mainly related to attention and executive functions [34] and these have been closely related to walking and mobility performance [35]. To this end, studies examining the cognitive differences between PD + FOG and PD − FOG have suggested that PD + FOG had worse executive, attentional, and visuospatial performance [36,37,38,39,40,41,42,43]. Furthermore, FOG severity was negatively correlated with performance on cognitive tests assessing executive functions, suggesting that FOG progression is associated with frontal lobe dysfunction [36,43]. This is also supported by neuroimaging studies suggesting a common pattern of grey matter atrophy between executive functions and FOG [44]. Additionally, PD + FOG performed worse than PD − FOG in dual task [40], that is the performance of two tasks simultaneously. Dual task is a common paradigm to test motor-cognitive interaction and a worse performance during dual task has been linked to reduced cognitive resources and lower attention and executive [45].

In PD, the coordination of bilateral stepping during gait quantified by the phase coordination index is reduced, especially in PD + FOG [18,46]. This result, however, is particularly marked during BW [18]. Gait coordination, rhythmicity, and asymmetry have been related to cognitive functions and proprioception; therefore, a more markedly increased gait asymmetry during BW could also be associated to the increase in attentional demands [20]. This is further supported by the finding that PD + FOG demonstrated more gait asymmetry than PD − FOG in BW under dual task conditions (Hackney and Earhart 2010).

This review has reported that during the gait cycle, PD + FOG decompose movement between joints more than PD − FOG [21]. This difference in decomposition indices between PD + FOG and PD − FOG could be related to the cerebellar involvement in FOG [47]. PD + FOG, indeed, have been reported to have an abnormal functional connectivity network of pedunculopontine nucleus affecting the corticopontine-cerebellar pathways and visual temporal circuits [47]. These alterations, in the context of a demanding walking task and increased gait variability (i.e., BW), might lead PD + FOG to decompose their movement patterns to improve their stability [21]. Plotnik et al. (2008) suggested that in patients with PD, when alterations in gait surpass a certain limit, this might trigger FOG. This limit could be regulated by the attention used by PD patients in gait tasks, environmental stressors, and postural stability.

During BW, compared to forward walking, individuals tend to decrease their speed, most likely due to the inability to see the gait direction [10]. Moreover, during BW in healthy participants, range of motion of the hip, knee, and ankle have been reported to be reduced compared to forward walking [10]. The same study considered the ankle joint as the main joint responsible for propulsion and shock absorption during BW [10]. The findings from our review are in line with these results. PD patients, indeed, markedly reduced their hip, knee, and ankle ROM during BW, most likely due to the reduced proprioception, and this is significantly higher in PD + FOG [7,20], supporting the idea that this subgroup of patients have a more marked alteration of proprioceptive inputs.

Finally, the worse performance and higher variability of PD + FOG compared to PD − FOG, mainly evident in stance and swing percent, could be explained, at least partly, by their longer disease duration (10.5 ± 1.00 years vs. 6.4 ± 0.57 years) and greater balance impairment (Berg Balance Scale score: 46.8 ± 0.85 vs. 50.0 ± 0.61) [9].

Taken together, the present findings support the use of BW in PD patients and particularly in the subgroup of patients with FOG. These patients, indeed, have already been reported to be more prone to falls and subsequent injuries [22]. Moreover, PD + FOG have been reported to have more severe disease progression and non-motor symptoms [48] and a higher disability with a lower quality of life [49].

The findings that, in this group of patients, several walking parameters linked to all walking domains (pace, rhythm, variability, asymmetry, and postural control [50] are altered during BW and that these alterations are more pronounced than in forward walking, support the implementation of BW during clinical and instrumented evaluation, as well as considering including this paradigm in mobility research and clinical trials evaluating walking and mobility in patients reporting FOG. In addition, the alterations observed during BW could be less responsive to dopaminergic therapy, thus making this assessment more stable and less influenced by pharmacological state [13].

### Limitations and Future Directions

The present study has some limitations. It was difficult to reproduce FOG in the laboratory [7,20]. Thus, the actual number of FOG episodes was unknown because FOG was rarely observed during the intervention [7]. Future studies that can detect FOG episodes in an actual daily environment are needed to understand the effect of FOG under different gait conditions [7]. The results are not generalizable due to the limited number of included studies and the small sample size therein [7,20,21]. Assessments were done during either the OFF-medication state [7,18,19,20] or the ON-medication state [9,17,21]. Thus, assessments in both states could be useful as FOG episodes mainly occur in the OFF-state and some in the ON-state [51]. There was a high number and heterogeneity of spatiotemporal and kinematic parameters in each article, thus, a strict comparison of results is challenging.

## 5. Conclusions

This systematic review summarized the literature about BW performance of PD patients with and without FOG. PD with FOG had worse BW performance than PD without FOG. PD with FOG walked slower with shorter stride and step than PD without FOG. Also, during the gait cycle, PD + FOG decompose joint movement more than PD − FOG. As well, PD + FOG demonstrated greater variability in gait, swing, and stance percent and showed worse stepping coordination. PD with FOG had greater reduction in ROM, mainly at the ankle joint than PD − FOG. This could be due to reduced proprioception and limited attention and cognitive resources from PD patients during a challenging mobility task, or due to altered cerebellar network and visuospatial processing. These results suggest that BW assessment could be relevant to better characterize PD patients and that this paradigm could add valuable information to clinical and instrumented evaluation as well as research and clinical trial.

## Figures and Tables

**Table 1:** The National Institutes of Health Quality Assessment Tool for Observational Cohort and Cross-sectional Studies.

		Question/Objective Clearly Stated	Population Clearly Defined	Participation Rate of Eligible Persons at Least 50%	Subjects Recruited from the Same Populations	Sample Size Justification, Power Description, or Variance and Effect Estimates Provided	Exposure(s) of Interest Measured Prior to the Outcome(s)	Timeframe Sufficient for an Association between Exposure and Outcome	Study Examines Different Levels of Exposure	Exposure Measures Clearly Defined	Exposure(s) Assessed More Than Once over Time	Outcome Measures Clearly Defined	Outcome Assessors Blinded	Loss to Follow-up after Baseline 20% or Less	Key Potential Confounding Variables Measured and Adjusted Statistically	Overall Rate
Hackney & Earhart 2009 [9]	Yes															Fair
	No															
	Other			NR									NA	NA		
Hackney & Earhart 2010 [17]	Yes															Fair
	No															
	Other			NR									NA	NA		
Myers et al., 2020 [21]	Yes															Fair
	No															
	Other												NR	NA		
Peterson et al., 2012 [18]	Yes															Poor
	No															
	Other			NR										NR		
Son et al., 2018 [20]	Yes															Fair
	No															
	Other			NR										NR	NR	
Son et al., 2022 [7]	Yes															Fair
	No															
	Other												NR	NA		
Sutter et al., 2017 [19]	Yes															Fair
	No															
	Other			NR										NA	NA	

NA: not applicable; NR: not reported.

**Table 2:** General information about the selected studies, including authors, year of publication, country, title, journal, and funding sources.

Author	Publication Year	Country	Title	Journal	Funding Sources	Study Design
Hackney and Earhart [9]	2009	USA	Backward Walking in Parkinson Disease	*Movement Disorders*	The American Parkinson Disease Association funded this work	Cross-sectional
Hackney and Earhart [17]	2010	USA	The Effects of a Secondary Task on Forward and Backward Walking in Parkinson’s Disease	*Neurorehabilitation and Neural Repair*	The American Parkinson Disease Association and NIH grant K01-HD048437.	Cross-sectional
Myers et al. [21]	2020	USA	Cross-sectional Analysis of Backward, Forward, and Dual Task Gait Kinematics in People With Parkinson Disease With and Without Freezing of Gait	*Journal of Applied Biomechanics*	National Institutes of Health (T32HD007434), the Greater St Louis Chapter of the American Parkinson Disease Association, and the American Parkinson Disease Association Center for Advanced PD Research at Washington University School of Medicine.	Cross-sectional
Peterson et al. [18]	2012	USA	Evidence for a Relationship between Bilateral Coordination during Complex Gait Tasks and Freezing of Gait in Parkinson’s Disease	*Parkinsonism and Related Disorders*	NIH TL1RR024995; NIH RO1HD056051-01; NIH 2T32HD007434-18A; Parkinson’s Disease Foundation; American Parkinson Disease Association Center for Advanced PD Research at Washington University.	Cross-sectional
Son et al. [20]	2018	Republic of Korea	Impacts of Freezing of Gait on Forward and Backward Gait in Parkinson’s Disease	*Gait & Posture*	The Dong-A University research fund	Cross-sectional
Son et al. [7]	2022	Republic of Korea	Turning Reveals the Characteristics of Gait Freezing Better than Walking Forward and Backward in Parkinson’s Disease	*Gait & Posture*	The Dong-A University Research Fund	Explorative
Sutter et al. [19]	2017	USA	Low to Moderate Relationships between Gait and Postural Responses in Parkinson’s Disease	*Journal of Rehabilitation Medicine*	NIH R01NS077959. K.S. is supported by the Clinical and Translational Science Award (CTSA) program of the National Center for Advancing Translational Sciences (NCATS) of the National Institutes of Health (NIH) at Washington University School of Medicine in St Louis (TL1 TR000449). R.D. is supported by NIH K12 HD055931.	Cross-sectional

**Table 3:** Demographic and anthropometric information of the participants included in each study.

Author, Year	Country	Sample Size (*n*)	Sex (M, F) (*n*)	Age, Mean (SD), Years	Disease Duration (SD), Years	Height, Mean (SD), m	Weight, Mean (SD), kg	BMI, Mean (SD), kg/m^2^	MDS-UPDRS III Score	H&Y Score
Hackney and Earhart 2009 [9]	USA	PD + FOG: 35 PD − FOG: 43	NM	NM	10.5 ± 1.00 6.4 ± 0.57	NM	NM	NM	NM	NM
Hackney and Earhart 2010 [17]	USA	PD + FOG: 35 PD − FOG: 43	NM	NM	10.5 ± 5.9 6.4 ± 3.7	NM	NM	NM	NM	NM
Myers et al., 2020 [21]	USA	PD + FOG: 13 PD − FOG: 31	M: 5, F: 8 M: 12, F: 19	64.2 ± 6.6 67.3 ± 9.2	NM	NM	NM	24.6 ± 3.0 23.8 ± 2.7	26 (13–48) 26 (3–49)	2 (2–3) 2 (1–2)
Peterson et al., 2012 [18]	USA	PD + FOG: 12 PD − FOG: 19	NM	72 ± 9 71 ± 9	8.0 ± 4.5 6.6 ± 5.1	NM	NM	NM	45.5 ± 15.2 41.6 ± 6.4	2.63 ± 0.83 2.37 ± 0.40
Son et al., 2018 [20]	Republic of Korea	PD + FOG: 10 PD − FOG: 16	M: 7, F: 3 M: 11, F: 5	70.24 ± 6.21 71.52 ± 6.34	symptom duration 4.60 ± 1.07 4.52 ± 1.23	1.63 ± 0.08 1.58 ± 0.01	60.99 ± 6.03 60.28 ± 10.24	23.0 ± 0.1 24.2 ± 0.1	35.95 ± 9.97 35.69 ± 7.60	2.60 ± 0.51 2.23 ± 0.59
Son et al., 2022 [7]	Republic of Korea	PD + FOG: 28 PD − FOG: 35	M: 18, F: 10 M: 14, F: 21	68.6 ± 5.5 70.5 ± 5.2	symptom duration 8.1 ± 5.1 4.5 ± 4.0	1.60 ± 0.01 1.55 ± 0.07	61.7 ± 9.5 57.8 ± 7.2	24.2 ± 0.1 24.1 ± 0.1	32.8 ± 8.9 33.6 ± 7.1	2.6 ± 0.4 2.4 ± 0.4
Sutter et al., 2017 [19]	USA	PD + FOG: 46 PD − FOG: 65	M: 28, F: 18 M: 37, F: 28	mean (95% CI): 67.9 (65.2, 70.5) 65.6 (63.2, 67.9)	median (IQR): 5.0 (3.0–9.5) 4.0 (1.3–7.5)	NM	NM	NM	41.5 (32.8–46.3) 34.0 (29.0–39.0)	I: 1, II: 33, III: 10, IV: 2 I: 3, II: 55, III: 6, IV: 1

M: male; F: female; *n*: number; NM: not mentioned; SD: standard deviation; BMI: body mass index; CI: confidence interval; IQR: interquartile range; MDS-UPDRS III: the Movement Disorder Society-sponsored revision of the Unified Parkinson’s Disease Rating Scale Part III; H&Y: Hoehn and Yahr scale.

**Table 4:** Description of the backward walking tasks.

Author, Year	Task	Length of Walkway	Number of Conditions	Walking Speed
Hackney and Earhart 2009 [9]	Instructions: Participants walked FW to accustom themselves to the mat, and then BW. Number of trials per condition: 3 trials (trials were averaged)	5 m instrumented computerized GAITRite walkway	2: FW BW	Normal pace
Hackney and Earhart 2010 [17]	Instructions: Participants performed simple conditions by FW and then BW. Next, participants performed dual conditions by FW while performing a mental arithmetic task aloud. This procedure was repeated while BW. Number of trials per condition: Simple conditions: 3 trials (trials were averaged) Dual conditions: 1 trial	5 m instrumented computerized GAITRite walkway	4: Single-task conditions: 2: FW BW Dual task conditions: 2: FW + mental arithmetic task aloud (counting backward from 100 by threes, from 50 by fours and from 75 by sixes) BW+ mental arithmetic task aloud (counting backward from 100 by threes, from 50 by fours and from 75 by sixes)	Normal or comfortable pace
Myers et al., 2020 [21]	Instructions: Participants walked a length of 7 m stretched diagonally across the capture volume in 3 conditions: FW, BW, and dual task. Number of trials per condition: Trials were grouped in blocks by condition (5 trials per block), and blocks were randomized (NM if trials were averaged)	7 m	3: FW BW Dual task (FW + list as many different words as they could that begin with a specified letter. The letter changed for every dual task trial)	Comfortable pace
Peterson et al., 2012 [18]	Instructions: Subjects completed the following 6 gait tasks in random order: FW, BW, turning to the left and right in a small radius circle and turning to the left and right in a large radius circle. Number of trials per condition: 5 to 8 trials for both FW and BW (NM if trials were averaged)	10 m	6: FW BW Turning to the left in a small radius circle Turning to the right in a small radius circle Turning to the left in a large radius circle Turning to the right in a large radius circle	Comfortable, preferred pace
Son et al., 2018 [20]	Instructions: During the FW and BW task, all patients wore Lycra shorts and a T-shirt, and completed the test with bare feet. Number of trials per condition: 3 trials (trials were averaged)	8 m	2: FW BW	
Son et al., 2022 [7]	Instructions: During FW and BW tests, participants were asked to walk at their preferred speed. During 360-degree turning test, participants were asked to turn at their preferred speed in both directions. Number of trials per condition: 3 trials (trials were averaged)	8 m	3: FW BW 360-degree turning test	Preferred pace
Sutter et al., 2017 [19]	Instructions: Participants wore shoes during evaluations and were allowed to rest as often as needed. Number of trials per condition: 3 trials (trials were averaged)	4.8 m GAITRite computerized walkway	2: FW BW	Preferred pace

**Table 5:** Outcomes of gait parameters computed in individuals with Parkinson’s disease with and without freezing of gait during backward walking.

Author, Year	Acquisition System	Gait Parameters	PD + FOG	PD − FOG	Differences between PD + FOG and PD − FOG (Absolute Difference; Percentage of Difference)
Hackney and Earhart 2009 [9]	5-m instrumented, computerized GAITRite walkway (CIR Systems, Inc., Havertown, PA, USA).	Spatiotemporal parameters:			
		Walking speed (m/s)	0.61 ± 0.05	0.73 ± 0.05	NSD (*p* = 0.091)
		**Stride length (m)**	**0.7 ± 0.04**	**0.8 ± 0.05**	↓ (0.1; −12.5%) (*p* = 0.032 *)
		Cadence (step/min)	114 ± 4.7	110 ± 3.5	NSD (*p* = 0.422)
		Base of support (m)	0.2 ± 0.01	0.2 ± 0.01	NSD (*p* = 0.321)
		**Functional ambulation profile**	**55.8 ± 2.2**	**64.1 ± 2.5**	↓ (8.3; −13%) (*p* = 0.027 *)
		**Swing (%)**	**30.3 ± 0.8**	**32.4 ± 0.6**	↓ (2.1; −6%) (*p* = 0.040 *)
		**Stance (%)**	**70.0 ± 0.9**	**67.7 ± 0.6**	↑ (2.3; 3%) (*p* = 0.031 *)
		Double support (%)	41.2 ± 2.3	37.8 ± 11.6	NSD (*p* = 0.065)
		**Variability of swing (%)**	**8.6 ± 3.2**	**6.4 ± 2.8**	↑ (1.8; 34%) (*p* = 0.013 *)
		**Variability of stance (%)**	**10.8 ± 3.0**	**5.7 ± 0.5**	↑ (5.1; 89%) (*p* = 0.010 *)
		Variability of stride length (m)	0.1 ± 0.01	0.1 ± 0.01	NSD (*p* = 0.325)
Hackney and Earhart 2010 [17]	5-m instrumented, computerized GAITRite walkway (CIR Systems, Inc, Havertown, PA)	Spatiotemporal parameters:	NM	NM	
		**Walking speed (m/s)**			↓ PD + FOG vs. PD − FOG (*p* ≤ 0.00625 *)
		**Stride length (m)**			↓ PD + FOG vs. PD − FOG (*p* = 0.004 *)
		Cadence (step/min)			NSD (*p* > 0.05)
		Base of support (m)			NSD (*p* > 0.05)
		**Functional ambulation profile**			↓ PD + FOG vs. PD − FOG (*p* < 0.001 *)
		Swing (%)			NSD (*p* > 0.05)
		Stance (%)			NSD (*p* > 0.05)
		Gait asymmetry			NSD (*p* > 0.05)
Myers et al., 2020 [21]	A Hawk Digital RealTime 8-camera system (Motion Analysis Corp, Santa Rosa, CA)	Spatiotemporal parameters:			
		**Timing of minimum ankle angle (% gait cycle)**	**95.2 ± 5.2**	**88.3 ± 8.4**	↑ (6.9; 8%) (*p* < 0.05 *)
		Timing of maximum ankle angle (% gait cycle)	87.7 ± 6.2	87.4 ± 5.0	NSD (*p* > 0.05)
		Timing of minimum knee angle (% gait cycle)	54.7 ± 8.4	51.4 ± 4.6	NSD (*p* > 0.05)
		Timing of maximum knee angle (% gait cycle)	39.7 ± 20.8	51.1 ± 18.7	NSD (*p* > 0.05)
		Timing of minimum hip angle (% gait cycle)	69.0 ± 3.7	69.6 ± 4.5	NSD (*p* > 0.05)
		Timing of maximum hip angle (% gait cycle)	52.8 ± 6.8	53.5 ± 5.0	NSD (*p* > 0.05)
		Kinematic parameters:			
		**Decomposition index hip–ankle**	**12.9 ± 8.2**	**8.8 ± 4.1**	↑ (4.1; 47%) (*p* = 0.03 *)
		**Decomposition index hip–knee**	**16.0 ± 8.3**	**10.2 ± 6.5**	↑ (5.8; 57%) (*p* = 0.03 *)
		Decomposition index Knee-ankle	12.5 ± 4.8	10.8 ± 7.5	NSD (*p* > 0.05)
		Hip ROM (°)	23.2 ± 6.8	25.0 ± 5.5	NSD (*p* > 0.05)
		Knee ROM (°)	42.0 ± 9.6	44.9 ± 9.7	NSD (*p* > 0.05)
		Ankle ROM (°)	22.9 ± 5.7	23.8 ± 4.7	NSD (*p* > 0.05)
Peterson et al., 2012 [18]	Six round footswitches (20 mm diameter, 1 mm thick; Motion Lab Systems; Baton Rouge, LA, USA)	Spatiotemporal parameters:			
		**Stepping coordination:**			
		**Phase coordination index**	**13.9 ± 3.9**	**10.9 ± 3.8**	↑ (3; 27%) (*p* = 0.01 *)
		**Temporal accuracy of steps**	**7.4 ± 3.2**	**5.3 ± 2.1**	↑ (2.1; 40%) (*p* < 0.001 *)
		**Consistency of steps**	**6.4 ± 1.4**	**5.6 ± 1.9**	↑ (0.8; 14%) (*p* < 0.001 *)
Son et al., 2018 [20]	The Plug-ingait model + 39 reflective markers. Six infrared cameras (Vicon, MX-T10, UK). Nexus software (version 1.83, VICON, UK).	Spatiotemporal parameters:			
		**Walking speed MAS, LAS (m/s)**	**0.46 ± 0.13**	**0.60 ± 0.14**	↓ (0.14; −23%) (*p* = 0.031 *)
		**Step length MAS (m)**	**0.22 ± 0.08**	**0.32 ± 0.09**	↓ (0.1; −31%) (*p* = 0.014 *)
		Step length LAS (m)	0.27 ± 0.05	0.32 ± 0.08	NSD (*p* = 0.123)
		**Step length AI**	**24.03 ± 19.20**	**11.03 ± 7.62**	↑ (13; 118%) (*p* = 0.030 *)
		Step time MAS (s)	0.49 ± 0.05	0.50 ± 0.07	NSD (*p* = 0.902)
		Step time LAS (s)	0.51 ± 0.04	0.51 ± 0.07	NSD (*p* = 0.946)
		Step time AI	5.06 ± 3.95	4.66 ± 2.18	NSD (*p* = 0.755)
		Kinematic parameters:			
		Hip ROM MAS (°)	26.64 ± 7.75	32.92 ± 8.53	NSD (*p* = 0.098)
		**Hip ROM LAS (°)**	**25.77 ± 7.39**	**33.76 ± 9.25**	↓ (7.99; −24%) (*p* = 0.048 *)
		Knee ROM MAS (°)	32.16 ± 8.84	36.55 ± 13.00	NSD (*p* = 0.404)
		Knee ROM LAS (°)	32.58 ± 10.62	41.34 ± 11.47	NSD (*p* = 0.088)
		**Ankle ROM MAS (°)**	**21.84 ± 5.60**	**30.43 ± 6.75**	↓ (8.59; −28%) (*p* = 0.006 *)
		**Ankle ROM LAS (°)**	**21.18 ± 3.31**	**27.67 ± 4.84**	↓ (6.49; −23%) (*p* = 0.003 *)
		**Toe clearance height MAS (cm)**	**2.10 ± 0.65**	**2.96 ± 0.94**	**↓ (0.86; −29%) (*p* = 0.031)**
		Toe clearance height LAS (cm)	2.75 ± 1.26	3.05 ± 1.01	NSD (*p* = 0.536)
		Maximum anti-phase (°)	8.35 ± 2.42	6.74 ± 2.50	NSD (*p* = 0.153)
Son et al., 2022 [7]	The Plug-ingait model + 39 reflective markers. Six infrared cameras (Vicon, MX-T10, UK). Nexus software (version 1.83, VICON, UK).	Spatiotemporal parameters:			
		Walking speed MAS, LAS (m/s)	0.44 ± 0.18	0.55 ± 0.17	NSD (*p* > 0.05)
		Stride length MAS, LAS (m)	0.47 ± 0.20	0.56 ± 0.15	NSD (*p* > 0.05)
		Stride time MAS (s)	1.03 ± 0.17	1.06 ± 0.13	NSD (*p* = 0.328)
		Stride time LAS (s)	1.03 ± 0.16	1.06 ± 0.14	NSD (*p* = 0.295)
		Cadence MAS (step/min)	119.78 ± 18.65	116.99 ± 17.45	NSD (*p* = 0.293)
		Cadence LAS (step/min)	118.84 ± 19.19	114.13 ± 15.05	NSD (*p* = 0.337)
		**Step length MAS (m)**	**0.25 ± 0.11**	**0.30 ± 0.07**	↓ (0.05; −17%) (*p* = 0.000 *)
		Step length LAS (m)	0.26 ± 0.09	0.30 ± 0.09	NSD (*p* > 0.05)
		**Normalization of step length MAS (m)**	**0.15 ± 0.07**	**0.20 ± 0.04**	↓ (0.05; −25%) (*p* = 0.000 *)
		Normalization of step length LAS (m)	0.16 ± 0.05	0.19 ± 0.05	NSD (*p* > 0.05)
		Step time MAS (s)	0.52 ± 0.09	0.52 ± 0.07	NSD (*p* = 0.381)
		Step time LAS (s)	0.51 ± 0.08	0.54 ± 0.07	NSD (*p* = 0.205)
		Kinematic parameters:			
		Hip ROM MAS (°)	27.67 ± 9.17	30.14 ± 9.80	NSD (*p* > 0.05)
		Hip ROM LAS (°)	27.61 ± 8.89	31.93 ± 9.24	NSD (*p* > 0.05)
		Knee ROM MAS (°)	33.28 ± 10.70	35.33 ± 12.32	NSD (*p* > 0.05)
		Knee ROM LAS (°)	34.87 ± 10.87	38.47 ± 10.68	NSD (*p* > 0.05)
		**Ankle ROM MAS (°)**	**21.70 ± 6.60**	**28.00 ± 7.40**	↓ (6.3; −22.5%) (*p* = 0.000 *)
		Ankle ROM LAS (°)	22.43 ± 6.05	27.74 ± 8.91	NSD (*p* > 0.05)
		Toe clearance height MAS (cm)	5.84 ± 1.23	6.20 ± 0.95	NSD (*p* > 0.05)
		Toe clearance height LAS (cm)	6.12 ± 1.39	6.29 ± 1.04	NSD (*p* > 0.05)
		Maximum anti-phase (°)	7.15 ± 3.38	6.94 ± 2.52	NSD (*p* = 0.329)
Sutter et al., 2017 [19]	4.8 m GAITRite computerized walkway (CIR Systems, Franklin, NJ, USA)	Spatiotemporal parameters:			
		**Walking speed (m/s)**	**0.64 (0.46–0.83)**	**0.87 (0.67–1.02)**	**↓ (0.13;** −28%) (*p* < 0.001 *)
		**Step length (m)**	**0.33 (0.24–0.44)**	**0.46 (0.39–0.55)**	**↓ (0.23;** −26%) (*p* < 0.001 *)

PD + FOG: PD with FOG; PD − FOG: PD without FOG; MAS: more affected side; LAS: less affected side; AI: asymmetry index; ROM: range of motion; NM: not mentioned; NSD: Non significant difference; *: significance. Significant results are highlighted in bold. ↓: decrease of values in PD + FOG compared with PD − FOG, ↑: increase of values in PD + FOG compared with PD − FOG.

**Table 6:** Distribution of spatiotemporal and kinematics backward walking parameters in included studies.

	Hackney and Earhart 2009 [9] (nPD + FOG = 35 nPD − FOG = 43)	Hackney and Earhart 2010 [17] (nPD + FOG = 35 nPD − FOG = 43)	Myers et al., 2020 [21] (nPD + FOG = 13 nPD − FOG = 31)	Peterson et al., 2012 [18] (nPD + FOG = 12 nPD − FOG = 19)	Son et al., 2018 [20] (nPD + FOG = 10 nPD − FOG = 16)	Son et al., 2022 [7] (nPD + FOG = 28 nPD − FOG = 35)	Sutter et al., 2017 [19] (nPD + FOG = 46 nPD − FOG = 65)	N (% of Articles)
Walking speed (m/s)								5 (71%)
Stride length (m)								3 (43%)
Step length (m)								3 (43%)
Cadence (step/min)								3 (43%)
Functional ambulation profile								2 (29%)
Base of support (m)								2 (29%)
Swing (%)								2 (29%)
Stance (%)								2 (29%)
Step time (s)								2 (29%)
Double support (%)								1 (14%)
Variability of stride length (m)								1 (14%)
Variability of swing (%)								1 (14%)
Variability of stance (%)								1 (14%)
Gait asymmetry								1 (14%)
Asymmetry of step length								1 (14%)
Normalization of step length								1 (14%)
Stride time (s)								1 (14%)
Timing of minimum hip angle (% gait cycle)								1 (14%)
Timing of maximum hip angle (% gait cycle)								1 (14%)
Timing of minimum knee angle (% gait cycle)								1 (14%)
Timing of maximum knee angle (% gait cycle)								1 (14%)
Timing of minimum ankle angle (% gait cycle)								1 (14%)
Timing of maximum ankle angle (% gait cycle)								1 (14%)
Phase coordination index								1 (14%)
Temporal accuracy of steps								1 (14%)
Consistency of steps								1 (14%)
Hip range of motion (°)								3 (43%)
Knee range of motion (°)								3 (43%)
Ankle range of motion (°)								3 (43%)
Maximum anti-phase (°)								2 (29%)
Toe clearance height (cm)								2 (29%)
Decomposition index (hip-ankle)								1 (14%)
Decomposition index (hip-knee)								1 (14%)
Decomposition index (knee-ankle)								1 (14%)

## Data Availability

Not applicable.
